# Nitrogen Fertilization Elevated Spatial Heterogeneity of Soil Microbial Biomass Carbon and Nitrogen in Switchgrass and Gamagrass Croplands

**DOI:** 10.1038/s41598-017-18486-5

**Published:** 2018-01-29

**Authors:** Jianwei Li, Chunlan Guo, Siyang Jian, Qi Deng, Chih-Li Yu, Kudjo E. Dzantor, Dafeng Hui

**Affiliations:** 1 Department of Agricultural and Environmental Sciences, Tennessee State University, Nashville, TN 37209 United States; 2 Jiangxi Provincial Key Laboratory for Bamboo Germplasm Resources and Utilization, Forestry College, Jiangxi Agricultural University, Nanchang, 330045 Jiangxi China; 3 Department of Biological Sciences, Tennessee State University, Nashville, TN 37209 United States

## Abstract

The effects of intensive nitrogen (N) fertilizations on spatial distributions of soil microbes in bioenergy croplands remain unknown. To quantify N fertilization effect on spatial heterogeneity of soil microbial biomass carbon (MBC) and N (MBN), we sampled top mineral horizon soils (0-15 cm) using a spatially explicit design within two 15-m^2^ plots under three fertilization treatments in two bioenergy croplands in a three-year long fertilization experiment in Middle Tennessee, USA. The three fertilization treatments were no N input (NN), low N input (LN: 84 kg N ha^−1^ in urea) and high N input (HN: 168 kg N ha^−1^ in urea). The two crops were switchgrass (SG: *Panicum virgatum L*.) and gamagrass (GG: *Tripsacum dactyloides L*.). Results showed that N fertilizations little altered central tendencies of microbial variables but relative to LN, HN significantly increased MBC and MBC:MBN (GG only). HN possessed the greatest within-plot variances except for MBN (GG only). Spatial patterns were generally evident under HN and LN plots and much less so under NN plots. Substantially contrasting spatial variations were also identified between croplands (GG > SG) and among variables (MBN, MBC:MBN > MBC). This study demonstrated that spatial heterogeneity is elevated in microbial biomass of fertilized soils likely by uneven fertilizer application in bioenergy crops.

## Introduction

Bioenergy crops are important alternative technology for sustainable replacement of fossil fuels^1,2^. A significant portion (over 30%) of biofuel plant biomass will come from dedicated energy crops such as the perennial switchgrass (*Panicum virgatum*) and gamagrass (*Tripsacum dactyloides* L)^3,4^. Nitrogen (N) fertilizers are widely used to increase yield of various bioenergy crops^5–9^. In bioenergy crop research field, soil microbial biomass is receiving increasing attentions due to its role in soil fertility and crop yield. Across various soil and plant types, intensive N fertilizations significantly alter soil microbial biomass and activities^10,11^. However, effects of fertilizations on spatial distributions of soil microbial biomass in bioenergy croplands remain unknown. Understanding effects of fertilization on soil microbial functionality, including spatial structures in various bioenergy croplands may enhance our ability to manipulate nutrient cycling *in situ* to maintain and improve soil quality, production sustainability and to adapt to climate change.

Intensive inorganic fertilizer inputs substantially restructure spatial heterogeneity of soil biogeochemical and microbial features at a variety of spatial scales^12–15^. Long-term cultivation with chemical N fertilizer amendments resulted in weak to moderate spatial heterogeneity of soil total nitrogen and phosphorus in 0–20 cm in the field plot to watershed scales^16,17^. Effects of mineral fertilizer inputs and the consequent spatial heterogeneity in soil pH exerted key controls on microbial biomass carbon and nitrogen contents as well as their spatial distributions in a long-term field trial of organic agriculture^15^. Despite lacking such information, indirect evidence supported strong correlation between spatial patterns of soil denitrifiers community with nitrate and other nutrients at scales relevant to land management^18^. Nevertheless, the altered spatial variation of soil microbial properties and structures is likely to affect the local distribution and abundance of plant species and the performance of individual plants and microorganisms^19^ and, therefore, to have consequences for both community structure and ecosystem-level processes^20–23^.

Although agricultural soils are generally more homogeneous than forests^14^, they show a substantial level of spatial variability with respect to soil biochemistry. Soil microbial biomass carbon (MBC) and nitrogen (MBN) exhibited moderate spatial dependence^24–26^. Röver and Kaiser^25^ reported that coefficients of variation of MBC and MBN can reach up to 44%. The spatial distribution of soil MBN demonstrated more hotspots than MBC, implying that MBN might be more sensitive to environmental disturbances^24^. Spatial structures and variations of bacterial community were found at surface and subsoil horizons at the microscale, and at the centimeter to meter scale^27^. At the ecosystem scale (>10 m), bacterial community composition and structure were subtly, but significantly, altered by fertilization, with higher alpha diversity in fertilized plots^28^.

Natural heterogeneity of microbial biomass and activity could be altered likely through soil chemical changes (e.g. pH) caused by fertilizer input^15^. Spatial patterns and the scale of soil variability differ markedly among edaphically similar sites and these differences are also conditioned likely by intensity and duration of fertilizations^13,14,29^. On the other hand, bioenergy crop species not only influence biomass yield^30,31^ but also spatial variations^25^. Plant trees play a major influence on spatial structures of soil microbial communities^32–34^. Among switchgrass and gamagrass, the latter possessed more significant root biomass and volume^35^ thus likely favoring nutrients scavenging and microbial activities and contributing to long-term spatial heterogeneity of soil microbial biomass^36–39^.

Taken together, previous results suggest that soil microbial features such as MBC and MBN can greatly respond to fertilization in their spatial variability compared with soils without fertilizer input for years in bioenergy croplands. The objective of this study is to investigate effects of N fertilization on spatial distribution of soil MBC, MBN and MBC:MBN in two bioenergy croplands (SG and GG) in a three-year long field experiment at Tennessee State University’s campus farm in Nashville TN, representing a typical bioenergy crop site in Middle Tennessee. Under no tillage or plowing and minor mechanical disturbance, N fertilizer input marked a major management practice in these research plots. We hypothesize that relative to soils that have never been fertilized for years, long-continued N fertilization re-structures spatial patterns of soil MBC, MBN and MBC:MBN at both croplands. The extent of altered spatial heterogeneity varies between variables (MBN > MBC) and crop types (GG > SG). We also explored whether there is significant correlation between soil pH and microbial variables. This study is expected to clarify the fertilization effect on redevelopment of spatial heterogeneity of key soil microbial features in typical bioenergy croplands.

## Material and Methods

### Site Description and Characteristics

This study was conducted at the Tennessee State University (TSU) Main Campus Agriculture Research and Extension Center (AREC) in Nashville, TN, USA (Lat. 36.12° N, Long. 36.98° W, elevation 127.6 m). Soil at this location is Armour silt loam soil (fine-silty, mixed, thermic Ultic *Hapludalfs*) with soil pH of 5.97 and organic matter content of 2.4% on average^40^. A field experiment was established with two crop types and three nitrogen (N) fertilization treatments in 2011 in a randomized block design^41^. The two crop types include no till cultivation of ‘*Highlander*’ variety of eastern ‘*Alamo*’ switchgrass (*Panicum virgatum* L.) and gamagrass (*Tripsacum dactyloides* L.). The switchgrass and gammagrass were abbreviated as SG and GG hereafter. The three N fertilization treatments include no N fertilizer input (NN), low N fertilizer input (LN: 84 kg N ha^−1^ in urea) high N fertilizer input (HN: 168 kg N ha^−1^ in urea). Each plot has a dimension of 3-m × 6-m and each treatment has four replicate plots.

### Soil collections and laboratory analysis

**Figure 1:**
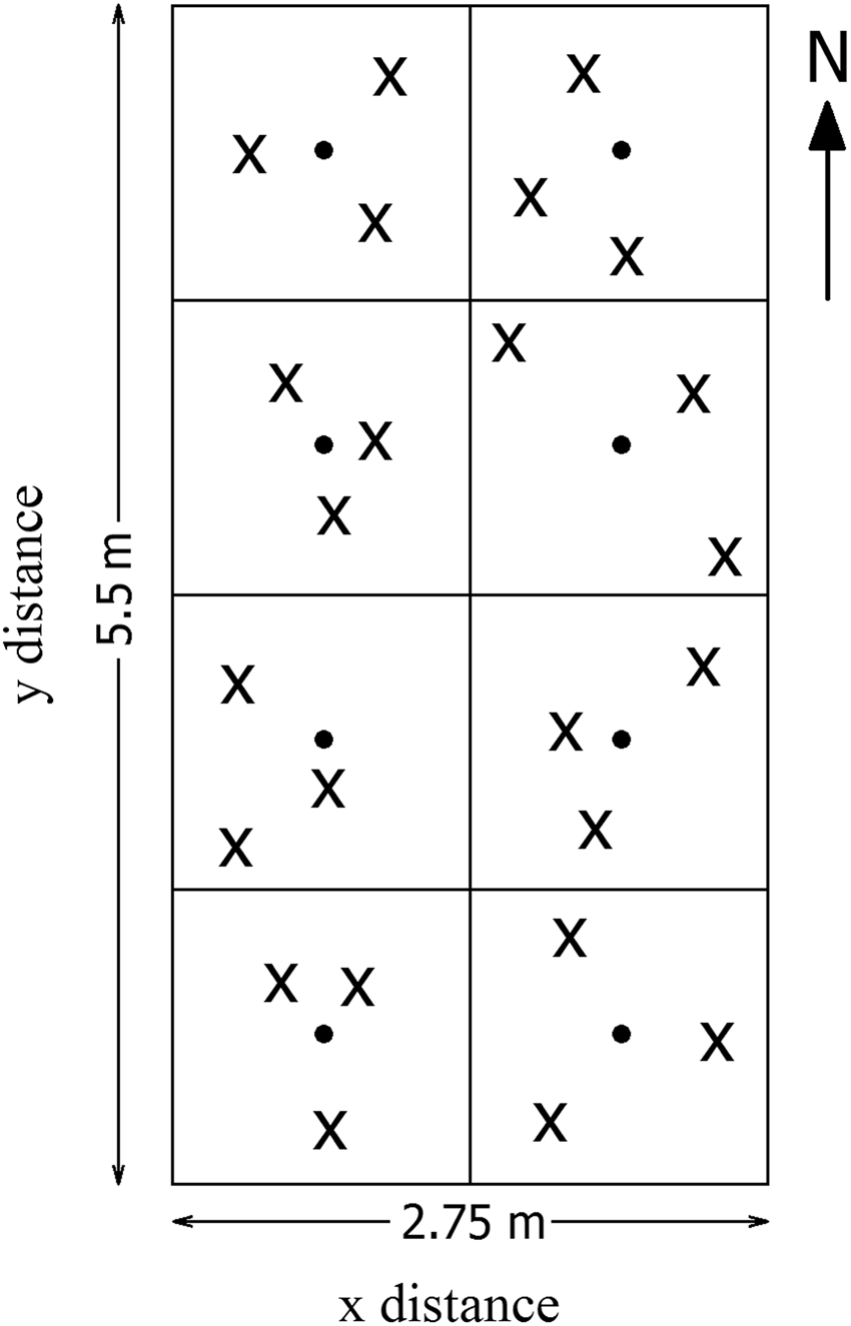
Illustration of a clustered random sampling design within a plot in the three-year long fertilization experimental site at the Tennessee State University (TSU) Agricultural Research Center in Nashville, TN, USA. Filled circles represent centroids (n = 8) and each plot consists of eight centroids with one in each sampling region (1.375 × 1.375 m). Xs represent sample locations determined from random directions and distances from a centroid. The extent of an interpolation map was thus determined by the minimum and maximum values at horizontal and vertical axes, and each map can attain its extent less than or equivalent to the study area (2.75 × 5.5 m rectangle).

On June 6^th^ 2015, soil cores were collected from 0 to 15 cm depth using soil auger (Thermo Fisher Scientific, Waltham, Massachusetts, USA) from 12 plots (2 crop × 3 N × 2 replicates). That is, two of the four replicated plots were selected in the current study. Within each plot, we identified a sampling area of 2.75-m × 5.5-m rectangle and the southwestern corner point was identified as the origin. Each plot was divided into two-square subplots and within each subplot, four centroids were identified and three cores were collected randomly given random direction and distance relative to each centroid (Fig. 1). When a soil core was collected, we recorded its location in reference to the origin taken as the southwestern corner, i.e. each sampling point had a unique x, y coordinates. Twenty-four cores were collected from each plot yielding 288 soil cores in 12 plots. All soil samples were transported to TSU lab in cooler filled with ice packs and subsequently stored at 4 °C until microbial analysis.

The visible roots and rocks were removed from soil cores by passing through a 2-mm soil sieve prior to microbial and chemical analysis. For each individual sample, soil gravimetric moisture content was determined by oven drying subsamples for 24 hours at 105 °C. Water extractable soil pH was measured given soil: water = 1:5^42^. The results were summarized in Table S1. Microbial biomass carbon and nitrogen were quantified as described in the following session. Besides, a composited subsample was produced by combining six soil samples of equivalent dry weight for each treatment. The air-dried subsamples were ground to a fine powder and sent to University of North Carolina at Wilmington Center for Marine Science for analysis of soil organic carbon content (SOC) and nitrogen content (TN).

Fresh soil samples (1.0 g) were used to estimate microbial biomass carbon (MBC) and microbial biomass nitrogen (MBN) in each core by chloroform fumigation-K_2_SO_4_ extraction and potassium persulfate digestion methods^43,44^. Briefly, 0.5 M K_2_SO_4_ was used to extract soil dissolved organic carbon and nitrogen from fumigated and unfumigated soil samples. Soil extracts were digested with 0.5 M K_2_S_2_O_4_ in oven at 85 °C for 20 hours. The K_2_SO_4_-extractable C and N in fumigated and unfumigated samples were determined by Shimadzu TOC-L & TNM-L (Shimadzu Corporation, Kyoto, Japan). MBC or MBN was calculated as the difference in K_2_SO_4_-extractable C or N concentration between fumigated and unfumigated soils, divided by 0.45 for C and 0.54 for N, respectively^42,45^. To minimize the variation likely induced due to unevenly soil mixing, laboratory tests were conducted and specific protocols were created to secure sufficient soil mixing. The variation of each measurement (i.e. coefficient variation) in multiple tests ranged from 2~8% based on our protocol.

### Statistical and geospatial analysis

Means, standard errors, and variances were estimated for MBC, MBN and their ratios in each plot. Frequency distributions were produced for each soil variable in each vegetation type after pooling all values of two replicated plots in the three nitrogen treatments. The two-way ANOVA was used to test whether means and coefficient of variance (CV) in MBC, MBN and their ratios differed significantly between fertilization treatments and crop species. To precede the ANOVA, the original data was log transformed if it violated equal variance assumption. The significance level is set at *P* < 0.05 and the analysis was conducted using R project^46^.

The Pearson moment correlation coefficients were derived between soil pH, MBC, MBN, and MBC:MBN. Cochran’s C test is used to test the assumption of variance homogeneity. The test statistic is a ratio that relates the largest empirical variance of a particular treatment to the sum of the variances of the remaining treatments. The theoretical distribution with the corresponding critical values can be specified^47–49^. Soil properties that exhibited non-normal distributions were log-transformed to better conform to the normality assumption of the Cochran’s C test^14^.

The study also derived the sample size requirement (*N*) in each plot given specified relative errors (γ, 0~100%) in order to evaluate how within-plot variances (i.e. sample size requirements) are altered by N fertilization or crop types at certain relative error.

1 $${\rm{CI}}=\bar{X}\pm {t}_{0.975}\times \frac{s}{\sqrt{n}}$$

2 $${\rm{\gamma }}=\frac{{t}_{0.975}\times \frac{s}{\sqrt{N}}}{\bar{X}}={t}_{0.975}\times \frac{CV}{\sqrt{N}}$$

3 $$\mathrm{ln}\,({\rm{N}})=2\times \,\mathrm{ln}({t}_{0.975}\times {\rm{CV}})-2\times \,\mathrm{ln}({\rm{\gamma }})$$

where CI, $\bar{X}$, *s*, *n*, *N*, *CV* and γ denote confidence interval, plot mean, plot standard deviation, sample number (n=24), coefficient of variation, sample size requirement and relative error, respectively. *t*_0.975_ = 1.96. The log transformed sample size requirement (*N*) has a negative linear relationship (i.e. slope = 2) with the log transformed relative error (γ).

In addition to the within-plot variance and derived statistics such as coefficient of variation and sample size requirement, the following geostatistical tools were used to quantify the spatial structure of soil microbial properties within and among plots. The methods were briefly described below and more details can be found in Li *et al*. (2010).

First, the trend surface analysis (TSA) is the most common regionalized model in which all sample points fit a model that accounts for the linear and non-linear variation of an attribute. The relationships between the soil properties and the x and y coordinates of their measurement location within the sampling plots are estimated with the trend surface model:

4 $$Soil\,property\,value={\beta }_{0}+{\beta }_{1}x+{\beta }_{2}y+{\beta }_{3}xy+{\beta }_{4}{x}^{2}+{\beta }_{5}{y}^{2}$$

The presence of a trend in the data was determined by the significance of any of the parameters β_1_ to β_5_, while the β_0_ term modeled the intercept^50,51^. Linear gradients in the x or y directions were indicated by significance of the β_1_ or β_2_ parameters. A significant β_3_ term indicated a significant diagonal trend across a plot. Significant β_4_ and β_5_ parameters indicated more complex, nonlinear spatial structure such as substantial humps or depressions. Trend surface regressions were estimated using *R* program^46^. Model parameters were determined to be significant at a level of *P* < 0.05.

Second, residuals from the trend surface regressions were saved for subsequent spatial analysis using a Moran’s I index^51^. The Moran’s I analysis^52–54^ was used to quantify the degree of spatial autocorrelation that existed among all soil cores taken from each plot. The resulting local Moran’s I statistics are in the range from −1 to 1. Positive Moran’s I values indicate similar values (either high or low) are spatially clustered. Negative Moran’s I values indicate neighboring values are dissimilar. Moran’s I values of 0 indicate no spatial autocorrelation, or spatial randomness. A significant autocorrelation is determined if the observed Moran’s I value is beyond the projected 95% confidence interval at certain distance. Correlograms for local Moran indices were estimated for each soil variable in each plot in a range of 0–5.5 meter with 0.25 meter incremental interval.

Third, due to relatively small sample sizes (n = 24) per plot^55^, we used inverse distance weighting (IDW) interpolation rather than ordinary kriging^56^. The maps produced by IDW offered direct and visual assessments from which to compare the spatial distributions of the soil properties among the plots. The IDW method derived maps was able to distinguish effects of different land uses on spatial distributions of soil biogeochemical features in South Carolina, USA^14^. The weights for each observation are inversely proportional to a power of its distance from the location being estimated. Exponents between 1 and 3 are typically used for IDW, with 2 being the most common^57^. Tests with different IDW exponents indicated that 2 was optimal with these data, as estimated values generated with an exponent of 2.0 showed the best fit with actual data in cross validation tests. ArcGIS 9.0 (ESRI, USA) was used to generate the IDW maps and perform cross validations.

## Results

### Central tendencies of soil biomass and pH under different treatments

**Figure 2:**
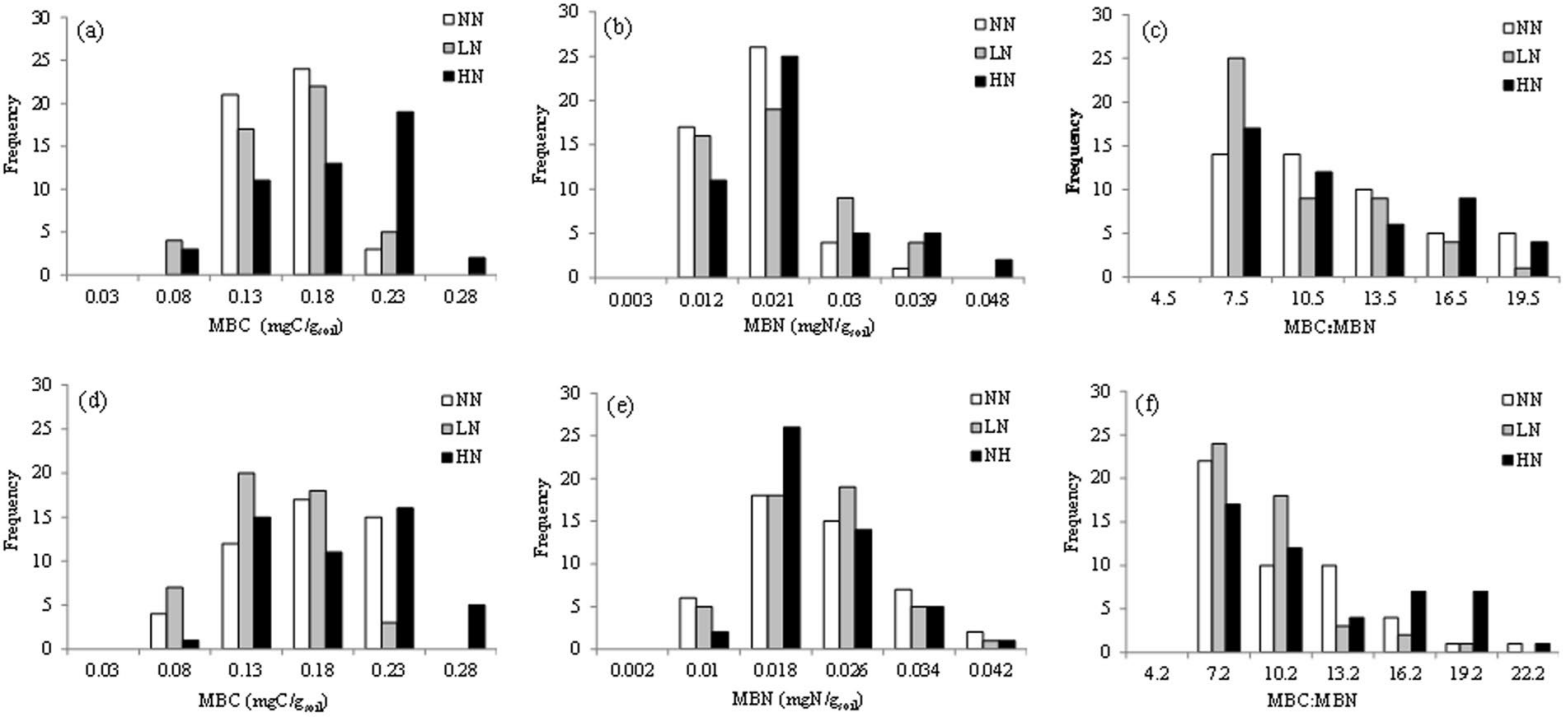
Frequency histograms of soil MBC, MBN concentrations and MBC:MBN under three fertilization treatments (i.e. NN, LN and HN) in SG (panels a~c) and GG (panels d~f) croplands in a three-year long fertilization experimental site at the Tennessee State University (TSU) Agricultural Research Center in Nashville, TN, USA. The number on the x-axis (i.e. 0.03, 0.08 in panel a) represents a range of (0, 0.03) and (0.03, 0.08), respectively. The abbreviations are referred to Table 1.

Mean MBC, MBN and MBC:MBN in NN treatment were not significantly different from that in either LN or HN for both SG and GG (Table 1). Mean MBC and MBC:MBN in HN treatment were significantly larger than that in LN treatment, but MBN was not significantly different between LN and HN treatments. These patterns were also reflected by the high frequency of larger MBC values and similar frequency of MBN among different N treatments (Fig. 2). The distribution of MBC:MBN showed higher frequency in lower values in general, but the frequency of higher values were particularly large for HN than LN for GG (Fig. 2). The average water extractable soil pH in different plots ranged between 5.91 and 6.12 (see Supplemental dataset), and showed neither significant differences between treatments nor significant correlations with any of microbial variables (Table S2).

**Table 1:** Mean (±SE) microbial biomass carbon (MBC, µgC/g_soil_), microbial biomass nitrogen (MBN, µgN/g_soil_) concentrations, MBC:MBN, and their respective coefficients of variance (CV, %) under three fertilization treatments (i.e. NN, LN and HN) in SG and GG cropland soils in a three-year long fertilization experimental site at the Tennessee State University (TSU) Agricultural Research Center in Nashville, TN, USA.

Crop	Fertilization	MBC CV(%)		MBN CV(%)		MBC:MBN CV(%)	
		μgC/g_soil_	%	μgN/g_soil_	%		%
SG	NN	137.4 ± 3.6^bc^	18.12	14.8 ± 0.7^a^	33.82	10.3 ± 0.6^a^	37.78
	LN	133.9 ± 5.3^c^	27.38	17.1 ± 1.0^a^	40.65	8.8 ± 0.5^ab^	39.33
	HN	160.8 ± 7.1^ab^	30.53	18.4 ± 1.3^a^	49.48	10.1 ± 0.6^a^	42.20
GG	NN	146.9 ± 6.7^abc^	31.58	18.6 ± 1.1^a^	41.38	8.8 ± 0.5^ab^	40.07
	LN	128.6 ± 5.0^c^	26.91	18.3 ± 1.0^a^	37.23	7.7 ± 0.4^b^	38.80
	HN	164.6 ± 7.1^a^	29.76	18.1 ± 0.9^a^	35.40	10.2 ± 0.7^a^	44.90

SG: switchgrass; GG: gammagrass; NN: No nitrogen; LN: Low nitrogen (84 kg N/ha/yr); HN: High nitrogen (168 kg N ha/yr). Different lowercase letters within each column represent significant difference between nitrogen fertilization treatments at *P* < 0.05 (N = 48). The units for MBC and MBN are mgC/g_soil_ and mgN/g_soil_ in other Tables and all Figures, respectively (1 mg = 1000 µg).

### Within-plot variance, within-plot CV and sample size requirement

The largest within-plot variances appeared consistently in one of the HN plots for all three variables in two croplands except for MBN in NN plot in GG (Table 2). Within-plot variances of soil MBC and MBN varied largely among different plots in SG but showed consistently high in GG. Cochran’s C test showed significantly different within-plot variances of MBC and MBN in SG but not in GG (Table 2).

**Table 2:** Comparison of the variances and Cochran’s C test results for soil MBC and MBN concentrations and MBC:MBN under three N fertilization treatments (i.e. NN, LN and HN) in SG and GG cropland soils in a three-year long fertilization experimental site at the Tennessee State University (TSU) Agricultural Research Center in Nashville, TN, USA.

Crop	Fertilization	Plot	MBC	MBN	MBC:MBN
SG	NN	P1	816.06	24.91	15.45
		P2	448.07	23.87	14.06
	LN	P1	1736.60	41.59	13.54
		P2	948.26	53.11	8.69
	HN	P1	**2073.30**	19.30	14.62
		P2	1129.91	**111.19**	**22.32**
Cochran’s test		*C* value	0.29	0.41	0.25
		*p*-value	0.04	0.00	0.23
GG	NN	P1	1089.91	**65.59**	6.35
		P2	618.26	54.00	13.28
	LN	P1	1007.95	43.80	8.07
		P2	809.07	47.35	9.83
	HN	P1	**1248.54**	51.56	**14.75**
		P2	769.38	26.78	3.00
Cochran^,^s test		*C* value	0.23	0.23	0.27
		*p*-value	0.61	0.58	0.12
Total Cochran^,^s test		*C* value	0.16	0.20	0.16
		*p*-value	0.03	0.00	0.06

Bold numbers in each column denote the first three largest variances than eleven, ten or nine plots, respectively at *p-value* < 0.05 (N = 24). The abbreviations are referred to Table 1.

**Figure 3:**
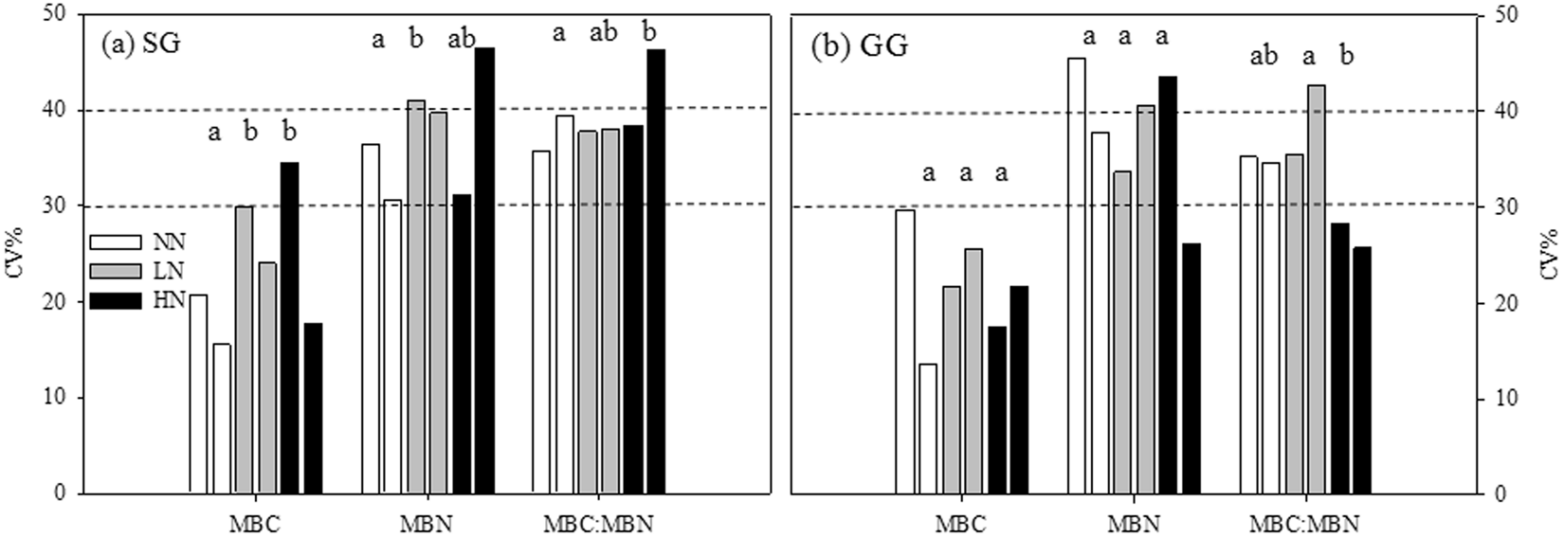
Within-plot CVs of MBC (mgC/g_soil_), MBN (mgN/g_soil_) concentrations, and MBC:MBN under three N fertilization (i.e. NN, LN and HN) in (**a**) SG and (**b**) GG cropland soils in a three-year long fertilization experimental site at the Tennessee State University (TSU) Agricultural Research Center in Nashville, TN, USA. The dashed lines represent a CV of 30% and 40%. The abbreviations are referred to Table 1. Different lowercase letters denote significant difference in CV between fertilization treatments for each variable at *P* < 0.05.

The within-plot CVs ranged from 15~48% for all variables and both croplands (Fig. 3). There is no significant difference of CV between two croplands (*P* > 0.05). The CVs under NN was significantly lower than that under LN or HN or both for three variables only in SG (*P* < 0.05). The CVs also differed significantly between LN and HN in GG (Fig. 3). The ranges of CVs for MBC were narrower than that for MBN and MBC:MBN. The largest CVs appeared to be in one of plots in HN treatment in SG, but none in GG (Fig. 3). As for the number of plots in twelve that produced CVs of more than 30% for MBN, MBC:MBN and MBC, respectively, they were six, six and one in SG, and five, four and none in GG. When the threshold of CV set at 40%, the numbers are two, one and none in SG, and three, one and none in GG.

**Figure 4:**
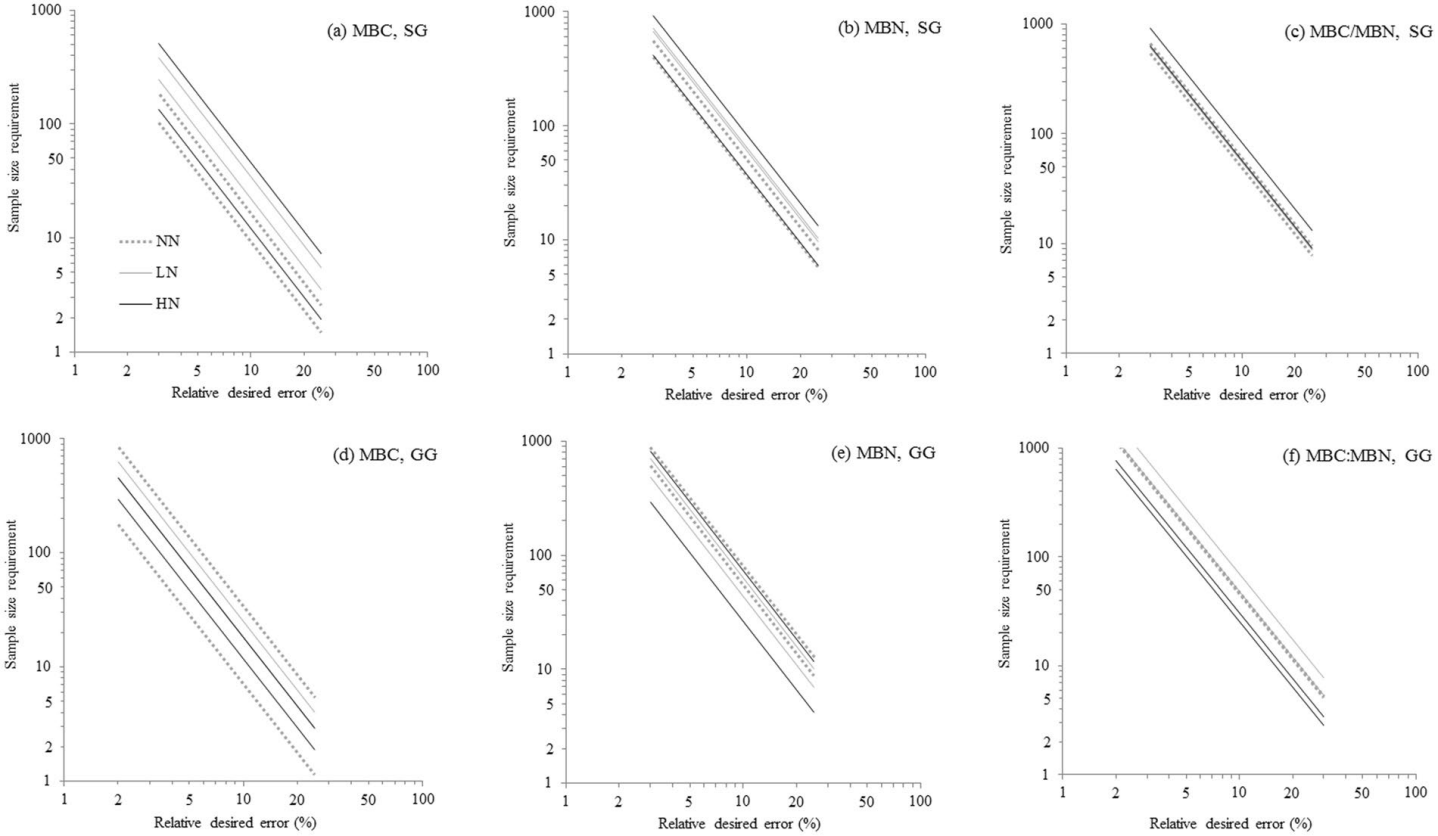
The relationship between log transformed sample size requirements and desired relative errors. Panels a~f denote the linear regression lines for soil MBC, MBN concentrations and MBC:MBN under three N fertilization treatments (i.e. NN, LN and HN) in SG and GG cropland soils in a three-year long fertilization experimental site at the Tennessee State University (TSU) Agricultural Research Center in Nashville, TN, USA. The log scale was applied on both axes. The abbreviations are referred to Table 1.

The plotted lines of sample size requirements were more widely separated for MBC than for MBN and MBC:MBN in both croplands (Fig. 4). Given the same relative desired error, much less sample size were required for MBC than for MBN and MBC:MBN in both SG and GG. The largest sample size requirements for all variables appeared to be at a HN plot in SG (Fig. 4a~c), but it is not true in GG (Fig. 4d~f). With such variability for soil MBN, even five samples per plot still will produce a relative error of the mean greater than ±30% (Fig. 4b,e), a sobering result given the level of interest in precise estimates of microbial dynamics. We think it not well appreciated that in either unfertilized or fertilized soils of gamagrass, the relative error of the estimate for MBN to be expected is >±50% if three samples are taken to estimate the mean (Fig. 4).

### Spatial heterogeneity under nitrogen fertilization and different crop species

Results of the trend surface analyses (Table 3) indicated that significant linear trends (i.e. diagonal direction) were identified in HN plots only (i.e. MBC at both SG and GG). More linear and nonlinear trends were identified in both LN and HN plots than that in NN plots, for MBC:MBN (SG and GG) and for MBN (GG only). The linear and nonlinear trends were identified for MBN in NN plot in SG only.

**Table 3:** Significant regression coefficients of trend-surface analysis and coefficients of determination (*r*^2^) for soil MBC, MBN concentrations and MBC:MBN under three N fertilization treatments (i.e. NN, LN and HN) in SG and GG cropland soils in a three-year long fertilization experimental site at the Tennessee State University (TSU) Agricultural Research Center in Nashville, TN, USA.

Crop	Variable	Fertilization	Plot	*β*_0_	*β*_1_x	*β*_2_y	*β*_3_xy	*β*_4_x²	*β*_5_y²	*r*²
SG	MBC	NN	P1	0.161***	—	—	—	—	—	0.407
			P2	0.131***	—	—	—	—	—	0.130
		LN	P1	0.229**	—	—	—	—	—	0.296
			P2	0.136**	—	—	—	—	—	0.253
		HN	P1	0.188**	—	—	—	—	—	0.290
			P2	0.200***	—	—	0.022*	—	—	0.289
	MBN	NN	P1	0.028***	−0.018*	—	—	0.006*	—	0.408
			P2	—	—	—	—	—	—	0.134
		LN	P1	0.0280*	—	—	—	—	—	0.152
			P2	0.0143*	—	—	—	—	—	0.545
		HN	P1	—	—	—	—	—	—	0.140
			P2	—	—	—	—	—	—	0.287
	MBC:MBN	NN	P1	—	—	—	—	—	—	0.092
			P2	11.443*	—	—	—	—	—	0.078
		LN	P1	—	—	—	—	—	—	0.105
			P2	11.157***	—	—	—	2.519*	0.544*	0.538
		HN	P1	16.528**	—	—	—	—	—	0.416
			P2	—	—	—	—	—	-	0.303
GG	MBC	NN	P1	—	—	—	—	—	—	0.184
			P2	0.226***	—	—	—	—	—	0.150
		LN	P1	0.162**	—	—	—	—	—	0.074
			P2	0.157**	—	—	—	—	—	0.097
		HN	P1	0.227***	—	—	—	—	—	0.153
			P2	0.138***	—	—	−0.010*	—	—	0.379
	MBN	NN	P1	—	—	—	—	—	—	0.176
			P2	—	—	—	—	—	−0.00278	0.346
		LN	P1	0.028**	—	—	—	—	—	0.210
			P2	0.041***	—	−0.0152***	—	—	0.0019**	0.582
		HN	P1	—	—	—	—	−0.0030*		0.346
			P2	0.015*	—	—	—	—	—	0.142
	MBC:MBN	NN	P1	10.379*	—	—	—	—	—	0.090
			P2	20.195***	−14.846*	—	1.591**	—	—	0.430
		LN	P1	—	—	—	—	—	—	0.304
			P2	—	—	1.537*	—		—	0.332
		HN	P1	13.969**	—	—	1.541*	—	—	0.414
			P2	8.503***	—	—	−0.720*	1.752*	—	0.485

*** and ***represent significance at P < 0.05, 0.01 and 0.001, respectively. The abbreviations are referred to Table 1.

**Figure 5:**
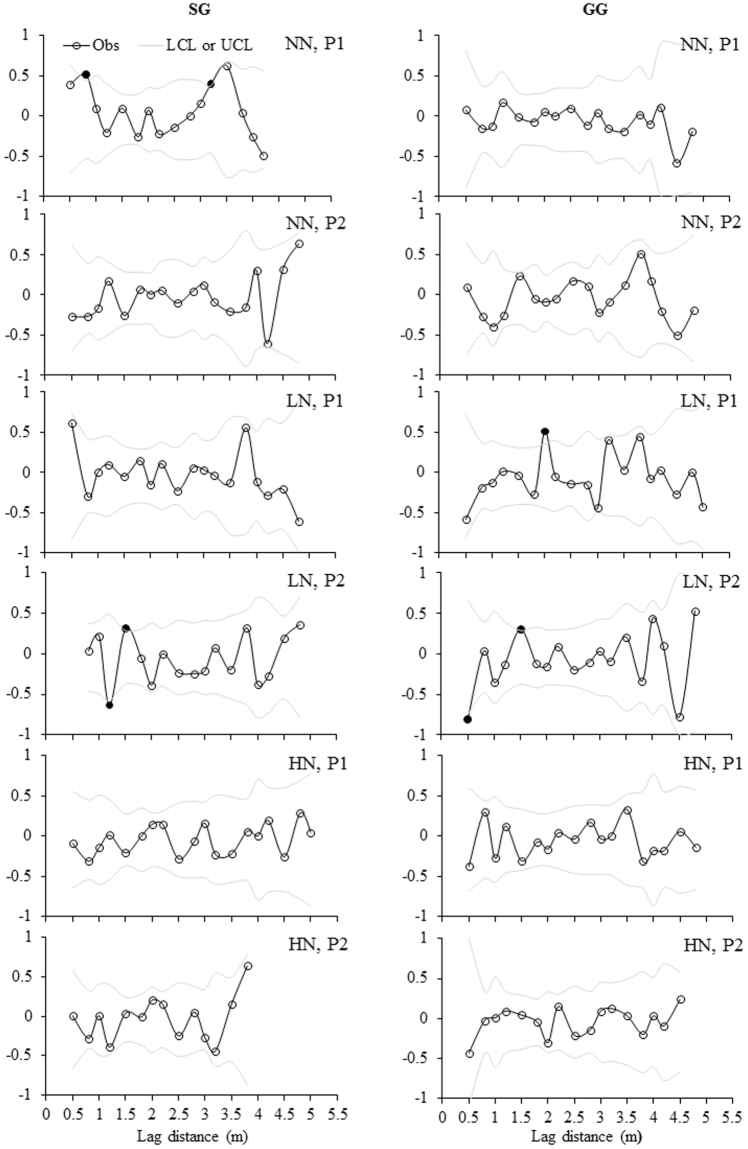
Moran’s *I* correlograms for soil MBC concentration under three N fertilization treatments (i.e. NN, LN and HN) in SG and GG cropland soils in a three-year long fertilization experimental site at the Tennessee State University (TSU) Agricultural Research Center in Nashville, TN, USA. Filled circles denote Moran’s *I* values that exhibited significant positive or negative autocorrelation. Obs: observations; LCL: low confident limit; and UCL: Upper confidence limit. Other abbreviations are referred to Table 1.

**Figure 6:**
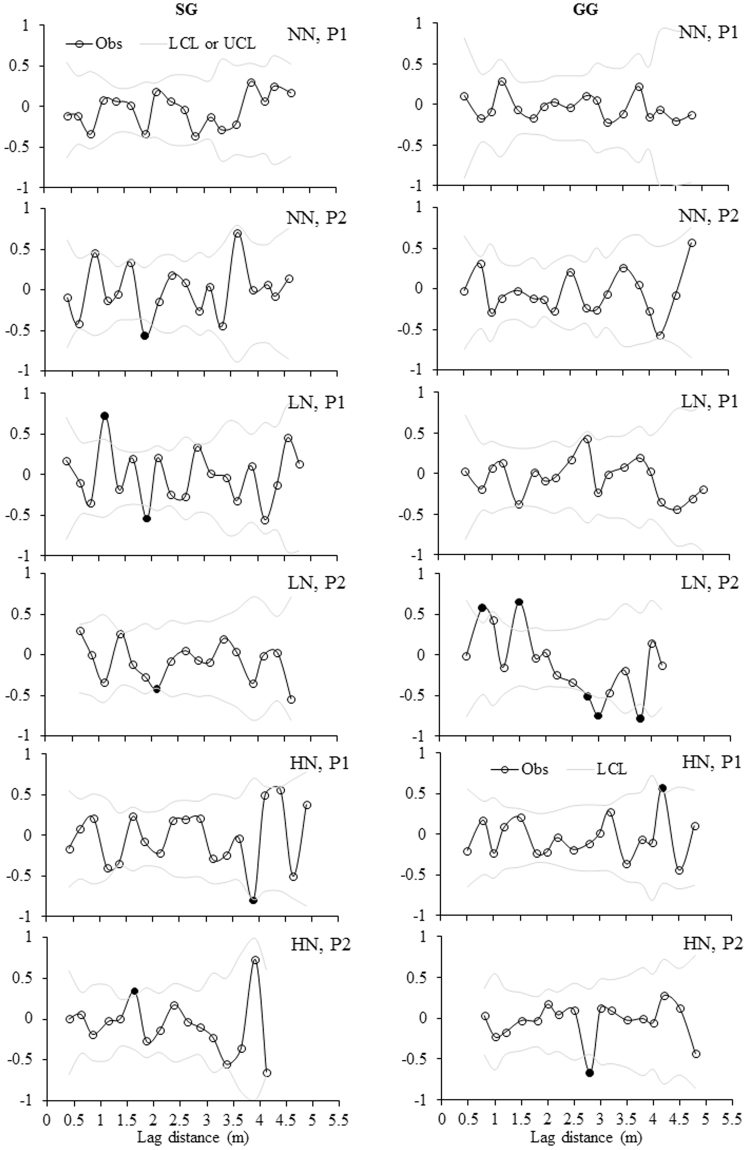
Moran’s *I* correlograms for soil MBN concentration under three N fertilization treatments (i.e. NN, LN and HN) in SG and GG cropland soils in a three-year long fertilization experimental site at the Tennessee State University (TSU) Agricultural Research Center in Nashville, TN, USA. Filled circles denote Moran’s *I* values that exhibited significant positive or negative autocorrelation. Obs: observations; LCL: low confident limit; and UCL: Upper conficence limit. Other abbreviations are referred to Table 1.

**Figure 7:**
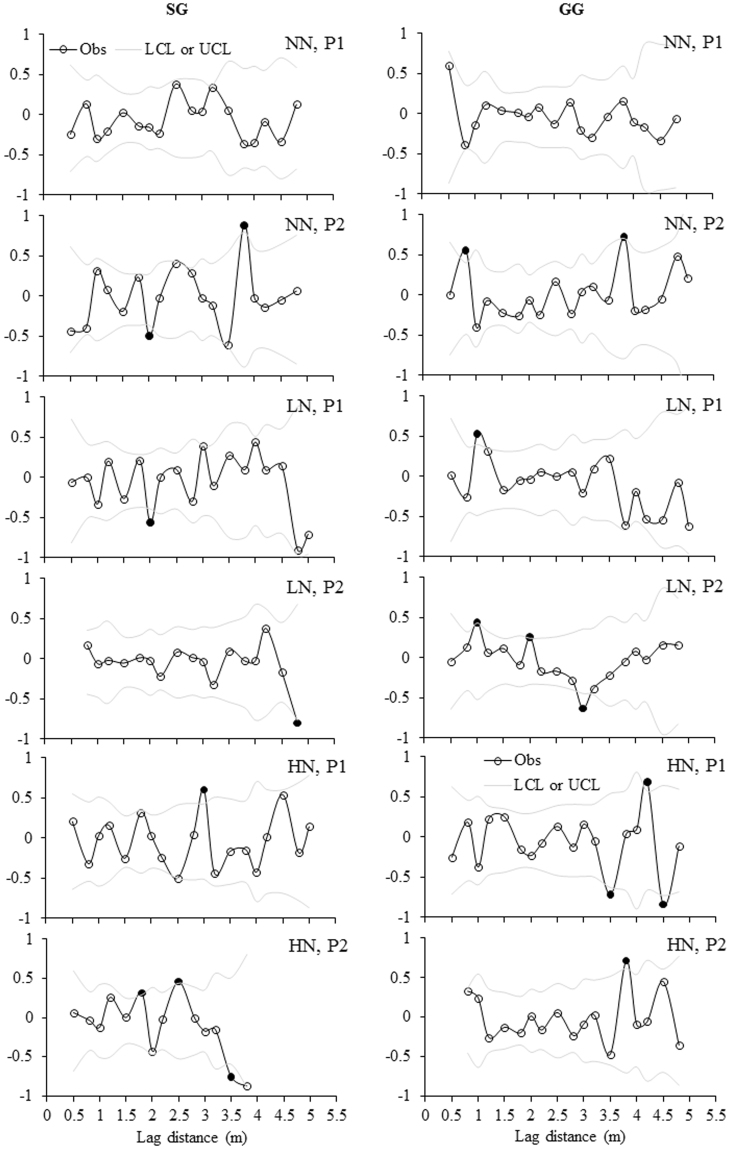
Moran’s *I* correlograms for soil MBC:MBN under three N fertilization treatments (i.e. NN, LN and HN) in SG and GG cropland soils in a three-year long fertilization experimental site at the Tennessee State University (TSU) Agricultural Research Center in Nashville, TN, USA. Filled circles denote Moran’s *I* values that exhibited significant positive or negative autocorrelation. Obs: observations; LCL: low confident limit; and UCL: Upper confidence limit. Other abbreviations are referred to Table 1.

Correlograms showed more significant autocorrelations of three variables in LN and HN than NN in either plot or treatment level, only except the equal number of significant autocorrelations of MBC in plots of NN and LN in SG (Fig. 5). For MBC, significant autocorrelations were present in each plot of LN treatment in GG (Fig. 5) and the lagging distances were either positive or negative ranging from 0.5 m to 2.0 m (Table 4). For MBN, only one significant autocorrelation was identified in one plot of NN in SG and none were present in NN plots in GG, whereas, such significant autocorrelations were present much more frequently in one plot of LN in GG (Table 4; Fig. 6). The lagging distances were either positive or negative for these significant autocorrelations and ranged from 0.75 m to 3.75 m (Table 4). For MBC:MBN, no significant autocorrelations were identified in one plot of NN in both croplands (Fig. 7) and one to three significant autocorrelations were present for other plots (Table 4).

**Table 4:** Summary of significant distance for spatial dependence based on Moran’s *I* values for MBC, MBN and MBC:MBN in 12 plots in a three-year long fertilization experimental site at the Tennessee State University (TSU) Agricultural Research Center in Nashville, TN, USA. The unit of the distance for spatial dependence is meter. The abbreviations are referred to Table 1.

Crop	Fertilization	Plot	MBC	MBN	MBC:MBN
SG	NN	P1	0.75, 3.25		
		P2		−1.75	−2.0, 3.75
	LN	P1		1.0, −1.75	−2.00
		P2	−1.25, 1.5	−2.25	−4.75
	HN	P1		−3.75	3.00
		P2		1.50	1.75, 2.50,−3.5
GG	NN	P1			
		P2			0.75, 3.75
	LN	P1	2.00		1.00
		P2	−0.5, 1.5	0.75, 1.5, −2.75, −3.0, −3.75	1.0, 2.0, −3.0
	HN	P1		4.25	−3.5, 4.25, −4.5
		P2		−2.75	3.75

**Figure 8:**
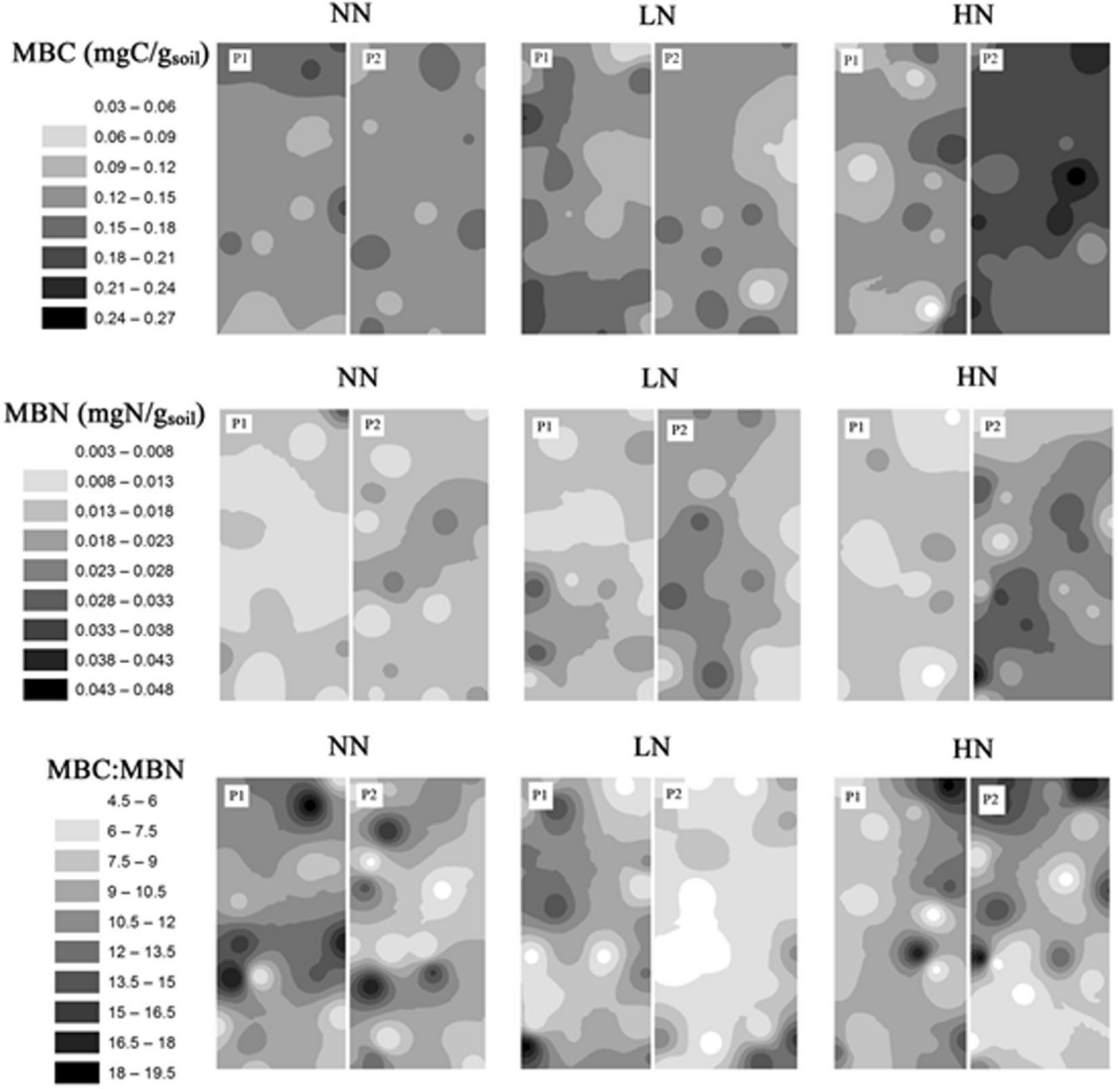
Spatial distribution of soil MBC and MBN concentrations and MBC:MBN under three N fertilization treatments (i.e. NN, LN and HN) in SG cropland soil in a three-year long fertilization experimental site at the Tennessee State University (TSU) Agricultural Research Center in Nashville, TN, USA. The interpolation maps were produced by inverse distance weighting (IDW) method. The abbreviations are referred to Table 1.

**Figure 9:**
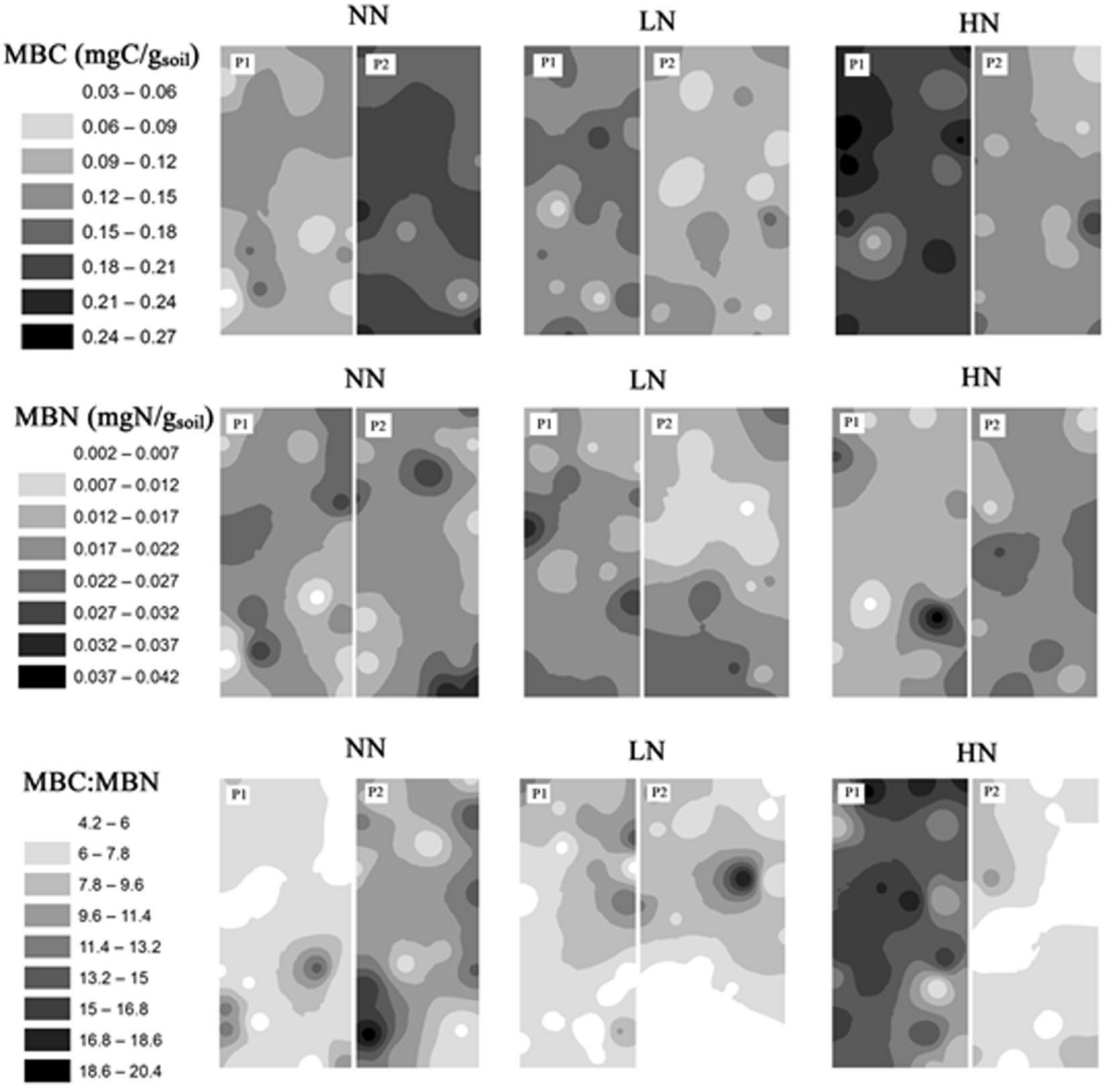
Spatial distribution of soil MBC, MBN concentrations and MBC:MBN under three N fertilization treatments (i.e. NN, LN and HN) in GG cropland soil in a three-year long fertilization experimental site at the Tennessee State University (TSU) Agricultural Research Center in Nashville, TN, USA. The interpolation maps were produced by inverse distance weighting (IDW) method. The abbreviations are referred to Table 1.

The IDW maps of within-plot patterns of MBC in LN or HN treatments, exhibited rather higher heterogeneity than those in NN treatments in switchgrass or gamagrass soils (Figs 8 and 9). There appeared to have greater within-plot heterogeneity of MBN in LN or HN treatments in SG cropland (Fig. 8), but less so in GG cropland (Fig. 9). It turned out to possess more hotspots of MBC:MBN than that of MBC and MBN across N treatments and croplands (Figs 8 and 9). Last, within-plot heterogeneity of all variables tended to be greater in GG cropland than that in SG cropland.

## Discussion

### N fertilization elevated spatial heterogeneity of soil microbial biomass

Similar to other important agricultural practices (i.e. plowing, mechanical disturbance), N fertilization is generally regarded to potentially homogenize the spatial distribution of soil chemical features in long-term cultivated lands particularly when compared with forests^14^. Despite very few studies examining effects of N fertilization on soil microbial features^27,32,58^, it is presumable that the high responsiveness of soil microbial features with disturbance^59,60^ could potentially override the general prediction of homogenization under N fertilizations. Based on a three-year fertilization experiment in two bioenergy croplands, this study revealed moderate spatial heterogeneity of soil microbial biomass C and N, and indeed, their spatial variations were generally elevated with low or high amount of N fertilizer input. In spite of great plot-plot variations within each fertilization treatment, different geostatistical approaches generally supported more linear and nonlinear surface trends and fine-scale spatial heterogeneity in the fertilized soils.

Possible explanations for the elevated spatial heterogeneity with N fertilizations may lie in the less complexity of management practice in the bioenergy croplands and the essentially high responsiveness of microbial properties in soils. First, the bioenergy croplands were subjected to continuous fertilization management over years but no plowing and less so in mechanical disturbance^61,62^. This unique management practice in the bioenergy croplands is distinct from the common practices implemented in conventional croplands such as wheat, corn and rice in many regions of world. Under common practices, the year- to decade-long plowing plus mechanical movement acted as the most significant driver that can physically and thoroughly mix and blend soil resources leading to homogenization^63^. Due to the cumulative effects induced by common practices other than N fertilization, spatial heterogeneity at various scales may be largely masked and turn to be subtle over long-term time period. Second, soil microbial communities and activities are highly responsive to soil nutrient availability such as nitrate and ammonium^10,64^, while N fertilizer inputs supply readily available nutrients and exert immediate influence on microbes over months to years^65,66^. This instant effect was also found evident at soil macroaggregate scale^67^. Therefore, the manual spread of N fertilizers in the field will likely lead to irregularity of nutrient deposit and clusters, and consequently favor the formation of hotspots in soil microbial communities^68^.

However, a more evident linear surface trend (i.e. diagonal direction) of MBN was identified in soils with no fertilizer input than fertilized soils, which likely attributed to the low sensitivity of this approach applied in the field plots at a scale of meter or broader^69,70^. Evidence showed the spatial autocorrelation of microbial properties in soil was well described at a scale of centimeter^69^, two magnitude of scale lower than meter. Consistent with the scale of centimeter, the study identified a unprecedentedly large number of significant autocorrelations, i.e. elevated fine-scale heterogeneity with fertilization in the same scenario. For instance, there are five significant Moran’s I values in one GG plot (i.e. P2 in LN) with the lagging distance ranging from 0.75 to 3.75 meter.

### Altered spatial heterogeneity with fertilization varied with crop types

Relative to SG, GG showed greater spatial variations in all three microbial variables such as more detectable linear and nonlinear surface trends, significant autocorrelations and hotspots. This is particularly true revealed by IDW maps that showed widespread appearance of hotspots in both MBC and MBN in GG, which is less evident in SG. The different spatial variations between the two crop species may be largely attributed to the root systems of two plants with contrasting characteristics. Roots are a key plant organs involved in resource competition and stand establishment^70^. Both GG than SG are warm-season grasses with a thicker and deeper rooting system. Their extensive root channels increased macropore flow in soil and consequently higher water infiltration rates^71^. They also exhibited enormous tolerance to low soil pH, aluminum toxic condition and high soil strength^35,72–74^. It is well known that plant root and microbes interact closely as mutualist^71^. The features of root morphology and physiology rendered plants capable of extending their access to large volume of soils for water, nutrients and resources thus favoring for soil microbial growth^71^. However, the strong effect of rhizosphere on relocating microbial niche may also differ between SG and GG because, though rarely compared quantitatively directly between the two plants, evidence pointed to the role of fine roots and its functions for switchgrass^75^ which is distinct from the more frequently reported much larger coarse roots and functions for gamagrass^71^. Though both SG and GG plantations resulted in strong clustering effects on spatial distribution of soil microbial communities, the larger size of roots for GG may play a key role in restructuring the larger patches of soil microbial biomass in soils, which is evident in GG than in SG.

### Altered spatial heterogeneity with fertilization varied among microbial variables

Across three fertilization treatments and two croplands, MBC showed relatively narrower within-plot variance and spatial heterogeneity than MBN or MBC:MBN. Also, MBC:MBN mimicked the spatial dependence of MBN, rather than MBC. In addition, the extent of elevated spatial heterogeneity due to fertilization was more pronounced for MBN or MBC:MBN than for MBC. These results collectively corroborated that MBN was a highly responsive variable in spatial dependence as compared with MBC revealed in several previous studies^24–26^. Due to nutrient poor conditions in these bioenergy croplands, the competitions for nutrients (e.g. N) between plant roots and microbes may be more intense given the widespread N limitations in terrestrial ecosystems^76,77^. On the other hand, the microbes with varying stoichiometry (i.e. C:N:P), that is, intrinsic N demand for growth and enzymatic kinetics^78,79^, may regulate their movement, colonization and growth given the soil indigenous N availability. This spatial assemblage of these microorganisms may be further complicated when N fertilizer granule manually applied in the field altered the distribution, diffusion and accessibility of readily available N forms, leading to more scattered hotspots of N island and more varied conditions of N hunger or limitation^80^. It is also likely that a more varied microbial biomass C:N may be driven by both altered physiological and compositional features in soil microbial community under N fertilizations^81^.

### Multiple drivers in restructuring spatial heterogeneity of microbial biomass

Despite the key role of soil pH on microbial growth and activity^82^ and bacterial community structure and diversity across environmental gradients^83,84^, no significant correlation was identified between soil pH and microbial biomass in our study (*p-value* > 0.05). The relatively uniform soil pH (5.7~6.3) across our study plots suggested the structured spatial heterogeneity of microbial biomass were likely driven by other edaphic factors (e.g. water availability), and management practices (e.g. fertilization means). Given the soil sampling conducted in dry summer season and a range of 15~18% gravimetric water content across plots, it is believed that the fertilization itself may overweigh the influence of other factors. In fact, in contrast to conventional cropland management with intensive plowing and mechanical disturbance, our research plots have not been plowed since it was established. Furthermore, fertilizers have been manually applied to soil by different people in our study plots. In addition, the relatively more pronounced spatial heterogeneity in GG than in SG indicates the influence of crop species on the spatial variations of soil microbial biomass. Our preliminary tests showed strong correlations and consistent spatial patterns between soil organic C (SOC), MBC and MBN (*p-value* < 0.05) in a few of our study plots. It suggests the possibly key control of SOC on MBC and MBN and highlights the keen need in the future to examine their relationships between two crops and under different N fertilization treatments. Therefore, a suite of interrelated edaphic, biochemical and management factors acted as major drivers during the redevelopment of spatial heterogeneity in fertilized soils of bioenergy croplands.

## Conclusions

Our study demonstrates that nitrogen fertilizer and the types of bioenergy crop type not only altered soil microbial properties’ central tendencies but also their spatial heterogeneities. In general, N fertilizations elevated the spatial heterogeneity of soil microbial biomass C and N as well as their ratio in both bioenergy croplands. Lacking the commonly applied agricultural practices such as plowing and mechanical disturbance, this study supported that in combination with edaphic and biochemical factors, intensive and uneven fertilizations tended to restructure the spatial heterogeneity of microbial properties, rather than to homogenize it. Substantially contrasting spatial variations were also identified between two bioenergy croplands (GG > SG) and among variables (MBN, MBC:MBN > MBC). Future researchers should better match sample sizes with the heterogeneity of soil microbial property (i.e. MBN) particularly in gamagrass cropland.

## Electronic supplementary material

Table S1&S2

Supplementary information
