# Supplementary material for: Nitrogen Fertilization Elevated Spatial Heterogeneity of Soil Microbial Biomass Carbon and Nitrogen in Switchgrass and Gamagrass Croplands
Source: Sci Rep. 2018 Jan 29;8:1734. doi: 10.1038/s41598-017-18486-5 (PMC5788856; doi:10.1038/s41598-017-18486-5)
Supplement: Supplementary file 1 — Table S1&S2 [file 41598_2017_18486_MOESM1_ESM.doc]

**Supplemental Materials**

**Nitrogen Fertilization Elevated Spatial Heterogeneity of Soil Microbial Biomass Carbon and Nitrogen in Switchgrass and Gamagrass Croplands**

Jianwei Li^1*^, Chunlan Guo^1,2^, Siyang Jian^1^, Qi Deng^3^, Chih-Li Yu^3^, Kudjo E Dzantor^1^, Dafeng Hui^3^

^1^ Department of Agricultural and Environmental Sciences, Tennessee State University, Nashville, TN 37209

^2^ Jiangxi Provincial Key Laboratory for Bamboo Germplasm Resources and Utilization, Forestry College, Jiangxi Agricultural University, Nanchang 330045, Jiangxi, China

^3^ Department of Biological Sciences, Tennessee State University, Nashville TN 37209

Table S1. Soil organic carbon (SOC) and nitrogen (TN) contents, stable C and N isotopic signatures, soil C:N and gravimetric moisture content in a three-year long fertilization experimental site at the Tennessee State University (TSU) Agricultural Research Center in Nashville, TN, USA.

| Crop | Fertilization | SOC | δ^13^C | TN | δ^15^N | C:N | Moisture |
| --- | --- | --- | --- | --- | --- | --- | --- |
|  |  | % |  | % |  |  | % |
| SG | NN | 1.24 | -24.48 | 0.11 | 3.93 | 11.01 | 18.4±2.1 |
|  | LN | 1.33 | -24.52 | 0.12 | 3.53 | 11.03 | 19.4±0.6 |
|  | HN | 1.32 | -24.76 | 0.13 | 2.44 | 9.89 | 17.4±0.2 |
| GG | NN | 1.31 | -24.95 | 0.13 | 2.67 | 9.95 | 18.0±0.4 |
|  | LN | 1.23 | -24.30 | 0.12 | 2.81 | 9.81 | 16.2±1.4 |
|  | HN | 1.34 | -26.70 | 0.13 | 2.70 | 10.11 | 17.0±0.3 |

Note: the number of soil samples is 2 for moisture and 1 for else, thus the standard error was presented for moisture. The abbreviations were referred to Table 1.

Table S2. Pearson moment correlation coefficients between microbial biomass carbon (MBC) and nitrogen (MBN), MBC:MBN and soil pH in a three-year long fertilization experimental site at the Tennessee State University (TSU) Agricultural Research Center in Nashville, TN, USA. Italic numbers indicate significant correlation coefficients at *p-value* < 0.05.

| Variable | MBC | MBN | MBC:MBN | pH |
| --- | --- | --- | --- | --- |
| MBC | 1.00 | *0.362* | *0.411* | -0.043 |
| MBN |  | 1.00 | *-0.64* | -0.021 |
| MBC:MBN |  |  | 1.00 | -0.037 |
| pH |  |  |  | 1.00 |
